# Supplementary material for: Epitranscriptomic N6-Methyladenosine Profile of SARS-CoV-2-Infected Human Lung Epithelial Cells
Source: Microbiol Spectr. 2023 Jan 10;11(1):e03943-22. doi: 10.1128/spectrum.03943-22 (PMC9927293; doi:10.1128/spectrum.03943-22)
Supplement: Supplemental file 4 — Supplemental material. Download spectrum.03943-22-s0001.pdf, PDF file, 0.02 MB [file spectrum.03943-22-s0001.pdf]

Epitranscriptomic *N*^{6}-methyladenosine profile of SARS-CoV-2-infected human lung epithelial cells

Stacia Phillips, Tarun Mishra, Shaubhagya Khadka, Dana Bohan, Constanza E. Espada, Wendy Maury, and Li Wu

Supplemental Tables (three separate Excel files)

Table S1: All differentially m^{6}a-modified transcripts in SARS-CoV-2-infected A549-hACE2 cells vs. mock-infected control cells.

Table S2: All pathways associated with at least one differentially methylated mRNA in SARS-CoV-2-infected A549-hACE2 cell vs. mock-infected control cells.

Table S3: Primer sequences used for qPCR amplification of selected targets.
